# Pharmacological Treatment for Neuroinflammation in Stress-Related Disorder

**DOI:** 10.3390/biomedicines10102518

**Published:** 2022-10-09

**Authors:** Dong-Hun Lee, Ji-Young Lee, Dong-Yong Hong, Eun-Chae Lee, Sang-Won Park, Yun-Kyung Lee, Jae-Sang Oh

**Affiliations:** 1 Department of Neurosurgery, College of Medicine, Soonchunhyang University, Cheonan Hospital, Cheonan 31151, Korea; 2 Soonchunhyang Institute of Medi-bio Science (SIMS), Soon Chun Hyang University, Cheonan 31151, Korea

**Keywords:** angiotensin II, angiotensin-converting enzyme, cyclooxygenase-2, glutamate, hypothalamic-pituitary-adrenal axis, microbiome, inducible nitric oxide synthase, neuroinflammation, peroxisome proliferator-activated receptor, reactive oxygen species, stress

## Abstract

Stress is an organism’s response to a biological or psychological stressor, a method of responding to threats. The autonomic nervous system and hypothalamic–pituitary–adrenal axis (HPA axis) regulate adaptation to acute stress and secrete hormones and excitatory amino acids. This process can induce excessive inflammatory reactions to the central nervous system (CNS) by HPA axis, glutamate, renin-angiotensin system (RAS) etc., under persistent stress conditions, resulting in neuroinflammation. Therefore, in order to treat stress-related neuroinflammation, the improvement effects of several mechanisms of receptor antagonist and pharmacological anti-inflammation treatment were studied. The N-methyl-D-aspartate (NMDA) receptor antagonist, peroxisome proliferator-activated receptor agonist, angiotensin-converting enzyme inhibitor etc., effectively improved neuroinflammation. The interesting fact is that not only can direct anti-inflammation treatment improve neuroinflammation, but so can stress reduction or pharmacological antidepressants. The antidepressant treatments, including selective serotonin reuptake inhibitors (SSRI), also helped improve stress-related neuroinflammation. It presents the direction of future development of stress-related neuroinflammation drugs. Therefore, in this review, the mechanism of stress-related neuroinflammation and pharmacological treatment candidates for it were reviewed. In addition, treatment candidates that have not yet been verified but indicate possibilities were also reviewed.

## 1. Introduction

Stress is an organism’s response to a physiological, biological, or psychological stressor, a method of responding to conditions such as physical and psychological threats [1]. Acute stress is a cause of psychological changes and causes loss of reality, anxiety, and arousal. On the other hand, chronic stress, although not as strong as acute stressors, can be more negative for health by affecting the body’s daily physiological responses over a long period of time. When a person is under chronic stress, permanent changes in physiological, emotional, and behavioral responses can occur [2]. However, most healthy individuals can still remain disease-free after facing a chronic stressful event, which can be a potential source of stress. This suggests that there are individual differences in the pathogenic effect. There are two main systems for controlling stress in humans or most mammals: the autonomic nervous system and hypothalamic–pituitary–adrenal axis (HPA axis) [3]. The sympathetic nervous system and the parasympathetic nervous system are antagonistic and regulate adaptation to acute stress [4,5,6]. The HPA axis regulates the release of cortisol, which has a great influence on body function, such as immunological function. Through these mechanisms, stress can affect memory, immunity, and metabolic diseases.

Chronic stress in humans can cause physiological, emotional, and behavioral changes in response, and severe acute and chronic stress can cause abnormalities in the serotonin system, catecholamine secretion, and major regulatory systems of the HPA axis [7]. Much evidence has been given that this stress can affect neuroinflammation, and interest in stress, neuroinflammation, and neurological diseases that result from it is increasing [8,9,10].

Inflammation is a protective reaction that helps to eliminate the original cause of cell damage and removes and repairs damaged cell and tissues [11,12,13]. Acute inflammation begins in macrophages and dendritic cells, which have receptors known as pattern regression receptors (PRRs), which recognize submolecules of pathogen-associated molecular pattern (PAMP) and damage-associated molecular pattern (DAMP) [14,15]. Acute inflammation occurs immediately after injury, and pro-inflammatory cytokine and chemokine call up neutrophil and macrophage to the inflammatory site. If this inflammatory reaction persists, chronic inflammation may persist and may develop into cardiovascular disease or chronic disease [16,17,18]. Chronic inflammation is a slow, long-term inflammation that lasts months to years. Infectious organisms remain in tissues for long periods of time or are associated with long-term inflammation-mediated diseases such as diabetes, cardiovascular disease, arthritis, and allergies. Stress and sleep disturbances are also risk factors for chronic inflammation, and mood disorders including chronic fatigue, insomnia, depression, and anxiety are common signs and symptoms that occur during chronic inflammation [19,20,21].

Neuroinflammation occurs when these inflammatory reactions affect the nerve tissue [22]. It may begin in response to infection, traumatic brain injury, toxic metabolites, or autoimmune signals [23,24]. Peripheral inflammatory cells can affect the brain through several mechanisms beyond the blood–brain barrier (BBB) [22,25,26]. In addition, neuronal inflammation can occur due to the activation of microglia and astrocyte in CNS [27,28]. The astrocytes are glial cells abundant in the brain and spinal cord. They perform many functions, including regulating cerebral blood flow, supplying nutrients to nervous tissue, maintaining extracellular ion balance, and post-infection and post-injury processes [29]. The astrocytes sense pro-inflammatory cytokines secreted by the CNS and peripheral immune cells recruited to the CNS and, in response, modulate the responses of neighboring cells throughout the CNS [27]. Activated nuclear factor kappa B (NF-κB) is central to astrocyte activation and contributes to the progression of CNS pathology [27,30]. Nuclear translocation of NF-κB in astrocytes was associated with TNF-α, IL-1β, 1L-17, ROS, Toll-like receptor (TLR), and other factors related to CNS inflammation [31,32]. The microglia are a type of glial cells, resident macrophages that act as a major form of immune defense in the CNS. As for the mechanism by which microglia actively regulate astrocyte function in neuroinflammation, it has been suggested that signaling induces astrocyte glutamate release in the presence of TNF-α [33]. It is a key cell in overall brain care, constantly cleaning out plaque, damaged or unnecessary neurons, synapses, and sources of infection. Astrocyte and microglia are innate immune cells in the CNS, which can over-generate pro-inflammatory cytokines when exposed to stress. Indeed, persistent stressors showed increases in granulocytes, natural killer cells, immunoglobulin A (IgA), and interleukin 6 (IL-6).

To treat this stress-related neuroinflammation, on the other hand, studies have shown that the induction of stress anxiety is suppressed when the release of monocyte from bone marrow and monocyte call-up to the brain are blocked. For example, antidepressants injected in mice prevented stress-derived neuroinflammation, and conversely, mice deficient in C-C chemokine receptor 2 (CCR2) and interleukin-1 receptor (IL-1R) did not experience anxiety after the repeated social defect (RSD) test.

Not only are stress and neuroinflammation related to each other, but the fact that stress reduction and anti-inflammation treatment can improve each other suggests the direction for the development of stress-related neuroinflammation drugs in the future. Therefore, in this review, the mechanism of stress-related neuroinflammation was studied, and pharmacological treatment candidates for it were reviewed.

## 2. Stress-Related Neuroinflammatory Mechanism

### 2.1. HPA Axis and Glucocorticoids

**Figure 1:**
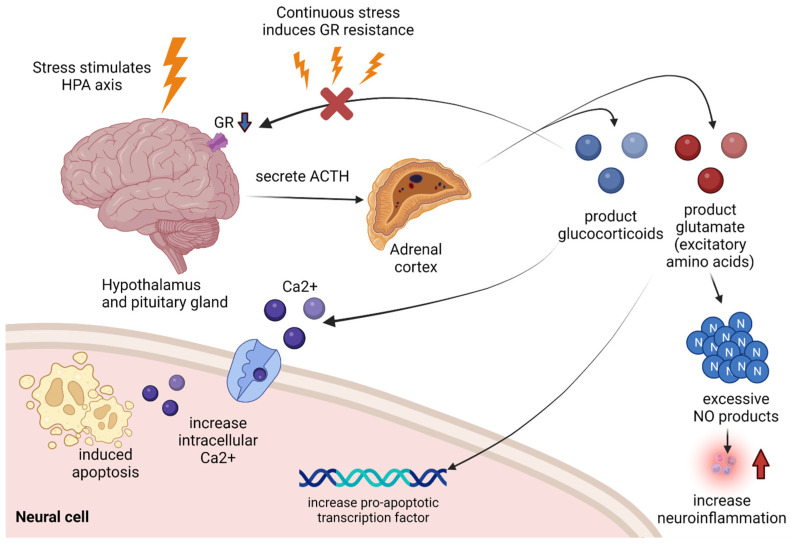
When the hippocampus is stimulated by stress, it secretes ACTH to stimulate the adrenal cortex, from which glucocorticoids and glutamates are overproduced. If a high level of glucocorticoids is maintained due to persistent stress, resistance occurs in GR, resulting in impaired glucocorticoids acceptance, which leads to impairment in anti-inflammatory action. In addition, it over-produces glutamate, an excitatory amino acid, which over-generates NO, resulting in NO-mediated neuroinflammation, and induces apoptosis in neural cells by upregulating the pro-apoptotic transcription factors. Glucocorticoids themselves also contribute to apoptosis by inducing intracellular calcium excess. The black arrow indicates that it contributes to the following response. The red arrow indicates increased neuroinflammation. ACTH: adrenocorticotropin hormone, GR: glucocorticoids receptor, HPA: hypothalamic-pituitary-adrenal, NO: nitric oxide.

The HPA axis is a complex interaction axis of the hypothalamic–pituitary–adrenal axis. It is a major neuroendocrine system that controls responses to stress and regulates many body processes [34]. Under stress, the hippocampus secretes corticotropin [35]. The corticotropin is adrenocorticotropic hormone (ACTH), which stimulates the adrenal cortex to induce the production of catecholamines and glucocorticoids in the systemic circulatory system. Therefore, high levels of glucocorticoids in blood plasma can be identified as typical characteristics of stress reactions. The glucocorticoids are activated by binding to glucocorticoid receptors (GR). The activated glucocorticoid complex regulates the expression of anti-inflammatory proteins and inhibits the expression of pro-inflammatory proteins [36]. However, if the stress state persists, glucocorticoid remains at a high level, resulting in fatigue in GR, which causes GR resistance, leading to high levels of glucocorticoid maintenance and impairment in anti-inflammatory action [37]. In addition, GR resistance causes a negative feedback failure, so the glucocorticoid level is not downregulated. As a result, stress-induced HPA axis hyperactivation leads to neuroinflammation (Figure 1) [7,38].

Some studies have shown that excessive production of stress-derived glucocorticoids damages the hippocampus and reduces long-term potentiation (LTP). As a mechanism for this, the hypothesis that changes in glucose carrier potential and decreases in mRNA levels cause problems in glucose transport in glial cells, resulting in problems in ATP production, is based on reduced ATP levels in the brain cortex of rats exposed to stress [39,40].

### 2.2. Glutamate (Excitatory Amino Acids)

Under stress conditions, excitatory amino acids increase rapidly, one of which is glutamate. The glutamate is used as a neurotransmitter for all major excitatory functions of the vertebrate brain and is involved in cognitive functions such as learning and memory in the brain because of its role in synaptic plasticity [41]. However, excessive glutamate accumulates outside the cell and can contribute to brain damage caused by neuroinflammation. The glucocorticoid described above increases intracellular calcium levels, causing glutamate to enter the cell through the NMDA glutamate receptor, resulting in nerve cell damage and eventual cell death, causing excitatory toxicity. In other words, increased intracellular glutamate concentration can induce apoptosis of nerve cells and nitric oxide (NO)-mediated neuroinflammation [42]. In addition, excessive glutamate mediates the promotion of pro-apoptotic transcription factors and the downregulation of transcription factors for anti-apoptotic genes [43]. Toxicity from excessive glutamate release and absorption disorders is associated with brain-related diseases such as stroke, autism, intellectual disability, and Alzheimer’s disease [44]. In fact, in the stressed rat model, it was confirmed that the excitatory amino acids transporters (EAAT) responsible for glutamate transport exhibited dysfunction, EAAT expression itself decreased, and glutamate aspiration decreased and remained at a high level [45,46].

### 2.3. Renin–Angiotensin System

**Figure 2:**
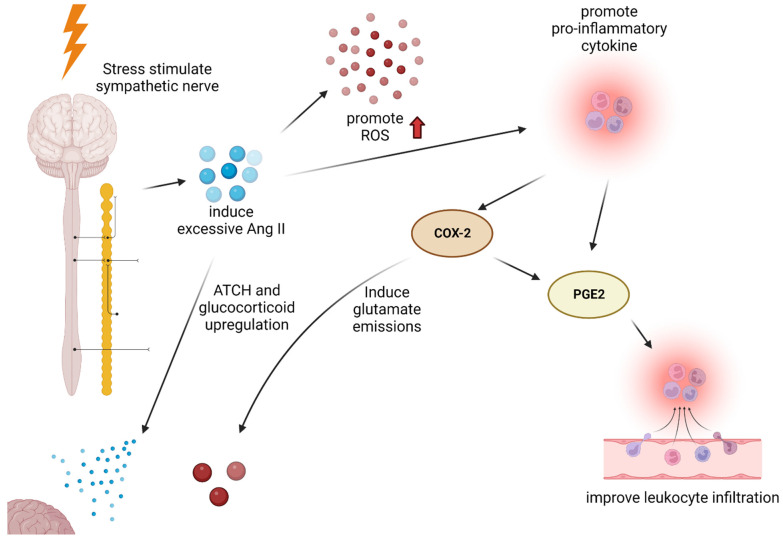
When stress stimulates sympathetic nerves, excessive Ang II is induced. It boosts ROS production and produces inflammatory cytokines. Pro-inflammatory cytokines, such as NF-κB, contribute to the production of COX-2 and PGE2. COX-2 is an important reactant contributing to the inflammatory response and can also contribute to neuroinflammation by inducing PGE2 activity by PGE2 RAS. PGE2 improves the infiltration of leukocyte. Ang II also contributes to ATCH and glucocorticoid upregulation, which may affect other mechanisms described above. The black arrow indicates that it contributes to the following response. The red arrow indicates an increase in ROS. ACTH: adrenocorticotropin hormone, Ang II: angiotensin II, COX-2: cyclooxygenase-2, PGE2: prostaglandin E2, ROS: reactive oxygen species.

Angiotensin II (Ang II) is an active principle of the renin–angiotensin system (RAS), originally involved in the regulation of blood pressure and body fluid metabolism, vasoconstriction, aldosterone release, sodium, and water retention and ingestion [47]. However, under stress conditions, the activity of Ang II can cause neuroinflammation. Acute stress upregulates Ang II production through sympathetic nerve activation of β-adrenergic receptors and increases levels of Ang II in the circulatory system [48,49]. These high levels of Ang II promote the production of reactive oxygen species (ROS) and pro-inflammatory cytokines [50]. The circulating pro-inflammatory cytokines, TNF-α and IL-1β, increase cyclooxygenase-2 (COX-2) activity and cause prostaglandin E2 (PGE2) production [51]. The PGE2, which occurs in response to physiological and psychological stress, is involved in several inflammatory pathways [52,53]. The PGE2 contributes to inflammation by improving leukocyte infiltration due to increased vascular projectivity when acting on receptors. As a result, Ang II causes neuroinflammation. In addition, increased circulation of Ang II can stimulate Ang II receptors in the pituitary anterior lobe and adrenal cortex, contributing to the upregulation of the pituitary anterior lobe, adrenocorticotropic hormone (ACTH), and glucocorticoids during stress [54,55]. It has been previously explained that glucocorticoids can contribute to neuroinflammation caused by stress (Figure 2).

### 2.4. Neuroinflammation by Stress-Related Oxidative and Nitric Oxide Products

Neuroinflammation can also be caused by oxidative and NO-mediated inflammatory reactions [56]. In the paragraph describing glutamate above, it was explained that an increase in glutamate induced by stress may cause NO-mediated neuroinflammation. Inducible nitric oxide (iNOS) is an enzyme that catalyzes NO production. The NO is involved in synaptic plasticity [57,58], smooth muscle relaxation [59], and vasodilation in CNS [60]. However, under sustained stress conditions, excessive production of NO is associated with a variety of diseases, along with causing neuroinflammation. Exposure to stress increases iNOS activity, expression at the brain and end of the body, and increases NO production [46,61,62]. Increased NO is also associated with NF-κB under stress. Stress-exposed rats caused NF-κB activation. When NF-κB inhibitors were administered just before stress, inhibition of iNOS expression, along with inhibition of NF-κB, was also shown, but the exact mechanism was not revealed [61]. The excessive NO production has also been proven to be associated with other diseases associated with neuroinflammation of the CNS, such as ischemic damage, Alzheimer’s disease, and Parkinson’s disease [63].

COX, also known as prostaglandin synthetase, is at the heart of anti-inflammation therapy for neuropathology, including neuroinflammation and neurodegenerative diseases. The COX has been identified as both COX-1 and COX-2 isoforms [64], among which COX-2 is rapidly expressed in multiple cells as a function of cytokines and pro-inflammatory molecules. In particular, COX-2 is prominently expressed in the hippocampus [65,66]. COX-2 is induced and expressed by macrophages and mononuclear cells involved in inflammation [67]. It is expressed in brain cells and is upregulated in various neurological diseases such as stroke and Alzheimer’s dementia [68]. It has also been explained through the renin–angiotensin system that COX-2 can biosynthesize prostaglandin to produce free radicals and activate PGE2 to contribute to neuroinflammation [67,69,70]. It can also contribute to neuroinflammation by inducing glutamate release and apoptosis from astrocytes rich in the brain [71]. The mRNA level of COX-2 was increased in mice who experienced forced swimming stress, and it was confirmed that it was mediated by glutamate and NF-κB [46,72].

## 3. NMDA Glutamate Receptor Inhibition for Treatment of Neuroinflammation

Upregulated glutamate due to stress can cause nerve cell damage and NO-mediated neuroinflammation due to excitatory toxicity. This is because glutamate is excessively introduced into the cell through the NMDA glutamate receptor, maintaining a high level of glutamate environment in the cell. The NMDA receptor is an ion channel protein that accepts glutamate in nerve cells. When glutamate and glycine are combined and activated, ions flow through the cell membrane, regulating cell signaling and synaptic plasticity by calcium, and are involved in memory cell activation [73]. The NMDA receptor is divided into the protein domain to which glutamate binds, the transmembrane domain responsible for transporting subunits, and the cytoplasmic domain directly involved in cell signal regulation.

It is possible to reduce oxidative and NO damage by inhibiting the NMDA glutamate receptor and preventing neuroinflammation and cell damage [74,75,76,77]. Competitive NMDA receptor antagonists to inhibit these NMDA glutamate receptors compete with the agent, glutamate, and bind to the same site of the receptor to block glutamate. However, this can cause problems because it also blocks the normal functioning of glutamate [78,79]. Blocking all NMDA receptor activity can lead to side effects, such as hallucinations and anesthesia. An alternative is the non-competitive NMDA receptor antagonist. Non-competitive NMDA receptor antagonists prevent excessive inflow of calcium. They block excess activity while preserving physiological NMDA receptor activity, and only blocks receptor ion channels when the channel is excessively opened [76].

The effectiveness of these NMDA glutamate receptors on neuroinflammation has been confirmed in animal experiments. Although increased TNF-α and increased activity of a TNF-α converting enzyme (TACE) in the brain cortex of rats that experienced stress, the administration of MK-801 (dizocilpine), a non-competitive NMDA receptor antagonist, reduced stress-derived activity, and expression of TNF-α [80].

## 4. Peroxisome Proliferator-Activated Receptor (PPAR) agonist for Treatment Neuroinflammation

A peroxisome proliferator-activated receptor (PPAR) is a protein that functions as a transcription factor that regulates gene expression [81]. PPAR plays an essential role in cell differentiation, development, and regulation of metabolism [82,83,84]. There are four types of PPAR: PPARα, PPARβ/δ, and PPARγ [83].

Among them, PPARα may exhibit an anti-inflammatory effect by inhibiting NF-κB activated in the PPAR-dependent pathway. In agonist studies acting as ligand on PPAR, it has been demonstrated to prevent multiple post-stress neuroinflammation and oxidative/NO damage, including iNOS inhibition, NF-κB blocking, TNF-α emission inhibition, and COX-2 expression reduction. In addition, PPARα activation plays an important role in the development of glucose homeostasis and insulin resistance as well as anti-inflammation, helping to reduce the risk of coronary heart disease and hypertension. As a ligand of PPARα, natural agents are typically omega-3 fatty acids.

PPARγ uses the prostaglandin mentioned earlier as ligand. It is expressed in microglia and astrocyte and is a target of anti-inflammatory activity in inflammatory nervous system diseases [85,86]. The expression of PPARγ was found to improve in the cortex of rats’ brains which underwent acute stress and was clearly identified in neurons and astrocytes [87]. When natural and synthetic PPARγ ligand was administered to rats, it was proven to prevent stress-derived CNS inflammation, and oxidation and nitrification reactions [88]. It can also activate antioxidant pathways such as nuclear factor erythroid 2-related factor 2 (NRF2) [89]. Synthetic agents include rosiglitazone, troglitazone, pioglitazone, and ciglitazone.

## 5. Angiotensin-Converting Enzyme (ACE) Inhibition and Angiotensin Receptor Blocking (ARB) for Treatment of Neuroinflammation

Ang II can cause neuroinflammation under stress [90]. It can increase pro-inflammatory cytokine and COX-2 activity, induce PGE2 production, and induce glucocorticoid secretion. Angiotensin 1 (Ang I) is a precursor of Ang II activated as a substrate of the RAS system. The ACE converts Ang I to Ang II [91]. ACE inhibitors can block this RAS system and contribute to improving neuroinflammation. It reduces ROS, decreases CRP that is upstream in the inflammatory response, and activates the complement system [92], and inhibits NF-κB. One clinical study suggested that ACE inhibitor and ARB-prescribed patients had a protective effect on stress-related disorders [93].

Some studies have shown that angiotensin(1–7) [Ang(1–7)] has the opposite effect of Ang II. The Ang II is hydrolyzed into Ang(1–7) through the action of ACE2. The Ang(1–7) binds and activates the Mas receptor to activate ACE2/Ang(1–7)/MasR axis, which weakens the hypertension induced by Ang II and weakens the inflammatory response [94]. Treatment with Ang(1–7) inhibited inflammatory markers in several disease models [95,96,97,98,99,100,101,102,103]. Angiotensin II receptor type 1 (AT1R) over-activates RAS by accepting Ang II [104,105,106,107]. It was shown that telmisartan, an AT1R blocker, reduced the synthesis of TNF-α and IL-1β of microglia, and significantly reduced iNOS [108]. Therefore, it contributes to beneficial effects as an ACE inhibitor and AT1R antagonist [109]. In addition, NF-κB was inhibited in the ischemic stroke rat model and cell death of white blood cells was induced [110,111,112].

## 6. Other Pharmacological Treatments for Neuroinflammation

In addition to the method of suppressing receptors, much effort is being devoted to developing pharmacological treatments to improve stress-related neuroinflammations. Several studies have also alleviated neuroinflammation by directly inhibiting inflammatory cytokines or by caring for pharmacological mental stress (Table 1).

NSAIDs are competitive inhibitors of COX, an enzyme that converts arachidonic acid to the inflammatory prostaglandin. It directly targets and inhibits COX-2 [113]. It has been found to be effective in inhibiting neuroinflammatory pathways and has helped improve symptoms in animal studies on depression, schizophrenia, affective disorder, and obsessive-compulsive disorder [114]. Studies have been conducted to support the association between chronic inflammation and mental disorders, which are reduced by the use of COX-2 inhibitors. However, in clinical trials, COX-2 inhibitors have been found to significantly increase the risk of heart attack and stroke, requiring a careful approach to use the drug, and some drugs have been revoked [115,116]. Typical COX-2 inhibitors include celecoxib, rofecoxib, and etoricoxib.

On the one hand, more and more studies are showing that antidepressants have immunosuppression and anti-inflammatory properties by regulating neurotransmitters [117]. According to some meta-analysis studies and clinical studies, antidepressant treatment showed a decrease in TNF-α, IL-1, IL-6, and a decrease in mRNA expression of both TNF-α and IL-1β in microglia in rats treated with antidepressants [118,119,120]. These improvements are explained by the discovery that antidepressant therapy induces upregulation of Treg cell, which can downregulate cytokine production [121].

## 7. New Candidates for Treatment

In addition to the drugs described in Table 1, efforts are being made to improve the neuroinflammation in stress-related disorders.

A study by Hanhai Zeng (2021) reported weakening of neuroinflammation with nuclear receptor binding factor 2 (NRBF2) [122]. The NRBF2 is involved in several diseases and stress conditions [123,124,125], especially autophagosome. Activation of autophagy reduces neuroinflammation [126].

There are also efforts using stem cells to alleviate neuroinflammation. Sandra A. (2015)’s study alleviated chronic inflammation of the stroke animal model with bone marrow stem cell therapy [127]. Jung (2016) also reported anti-inflammatory properties of CNS with mesenchymal stem cell therapy [128]. Several other studies have shown that stem cell therapy appears to help alleviate neuroinflammation. However, publications suggest that further investigation is needed in preparation for side effects commonly found in stem cells.

A relatively recent study is also paying attention to microbiome. This axis, called “gut–brain axis”, indicates that the health of the brain and gut is deeply related. Importantly, this gut microbiome (GM) imbalance can affect neuroinflammation in the brain. Sudo (2004)’s study investigated the effect of GM on HPA axis reactions on stress and found that HPA axis reactions were over-activated in mice with unbalanced GM than those with normal GM [129]. In addition, GM imbalances can transform the expression of tight junction proteins that maintain the BBB’s integrity, destroying the BBB’s integrity and helping to migrate inflammatory cells. Indeed, in preclinical studies, it has been reported that GM-deficient mice easily destroy the BBB permeability by regulating claudin-5 and occluding expression [130,131,132]. However, there are not enough studies using GM as a strategy to treat neuroinflammation at present. There have been several animal model studies attempting to intervene in GM composition, but they have not been followed in the long run [133,134]. Although not a pharmacological treatment, diet such as intermittent fasting can cause long-term changes in GM composition. Calorie restriction through intermittent fasting can positively affect GM composition in favor of the growth of beneficial anti-inflammatory microbial systems.

## 8. Conclusions

The brain was once considered to have immune privileges, but research into neuroinflammation and inflammatory cells passing through the BBB is now widely known. In addition, neuroinflammation in CNS is activated not only by response to infection or trauma, but also by psychological stress. Stress stimulates the HPA axis to upregulate glucocorticoids and induce the release of excitatory amino acids, such as glutamate. Continuous stress induces resistance of GR, which hinders the anti-inflammatory action of glucocorticoids. Elevated glutamate induces apoptosis due to excessive intracellular concentration of calcium and contributes to NO production, resulting in NO-mediated neuroinflammation. In fact, a study on rats identified elevated EAAT and glutamate levels and found that they were associated with many neurological diseases. Ang II is hyperactivated from sympathetic nerves under stress conditions, inducing ROS and pro-inflammatory cytokines. It can also contribute to the upregulation of ATCH and glucocorticoids mentioned above, which can also affect neuroinflammatory induction by other mechanisms. Oxidative/NO products produced through these processes are associated with NO activity and stimulate COX-2 activity.

Because each of these mechanisms is hyperactive through the receptor, an antagonist to the receptor can be intervened to improve stress-derived neuroinflammation. In fact, in many studies using animals, NMDA glutamate receptor antagonists reduced the activity of TNF-α, reduced oxidative/NO damage of astrocytes and neurons with PPAR agents, and activated antioxidant mechanisms such as NRF2. ACE inhibitor also inhibited inflammatory activity such as COX-2 in clinical and animal studies. However, the improvement effect has been confirmed, but the mechanism is not clear, and excessive blocking of the receptor may interfere with normal functions, so continuous research is needed.

In addition, it is very interesting that stress-induced neuroinflammation can be responded to with anti-inflammatory treatment, and pharmacological treatment methods that treat psychological stress, such as antidepressants, can lower the level of neuroinflammation. Indeed, treatment of neuroinflammation with antidepressants showed decreased expression of pro-inflammatory cytokines, such as TNF-a, IL-1, and IL-6, in experimental animals. Therefore, it is expected that research will become active on whether not only direct pharmacological treatments for neuroinflammation, but also pharmacological or psychological treatment methods that improve psychological stress, can help to improve neuroinflammation.

Moreover, beyond well-known pharmacological treatments, neuroinflammation treatment using NRBF2, stem cell therapy, and GM balance has not been studied well in the past, but it is expected to be a new treatment strategy in the future. In particular, since GM also affects the HPA axis and BBB, which are the main mechanisms of stress-related inflammations, further investigation is expected.

## Figures and Tables

**Table 1:** Various pharmacological treatments for the treatment of neuroinflammation drugs.

Types of Treatments	Mechanism	Typical Drugs
**ACE inhibitor and ARB**	Inhibits converting enzymes that activate Ang II, or converts Ang II into Ang(1–7), which exhibits an opposite effect	Benazepril, captopril, enalapril, lisinopril, perindopril, ramipril, trandolapril
**Antidepressant**	Stress suppression, or up-regulation of Treg cells that limit cytokines	Amitriptyline (*Elavil*), fluoxetine, imipramine, paroxetine
**COX-2 inhibitor**	Direct inhibition of COX-2, which is important for inflammatory reactions, improves neuroinflammatory pathways, and improves depression	celecoxib, etoricoxib, rofecoxib
**NMDA receptor antagonist**	Reduces oxidative and NO damage by inhibiting excessive glutamate in cells and improves neuroinflammation	dizocilpine
**PPAR agonist**	Prevents various post-stress neuroinflammatory pathways by inhibiting NF-κB, TNF-α, COX-2 and iNOS when injecting agents.	ciglitazone, rosiglitazone, troglitazone, pioglitazone

ACE: angiotensin-converting enzyme, ARB: angiotensin receptor blocker, COX-2: cyclooxygenase-2, iNOS: induced nitric oxide synthase, NO: nitric oxide, TNF-α: tumor necrosis factor-α.

## Data Availability

No new data were created or analyzed in this study. Data sharing is not applicable to this article.
